# Dupilumab-associated anti-glomerular basement membrane nephritis in a patient with eosinophilic granulomatosis with polyangiitis: a case report

**DOI:** 10.1186/s12882-025-04500-w

**Published:** 2025-10-07

**Authors:** Kei Nakada, Reika Flora Moriya, Kenichi Tanaka, Hiroki Ejiri, Fumiya Kitaguchi, Naoya Suto, Guy Watanabe, Hiroshi Kimura, Mizuko Tanaka, Junichiro James Kazama

**Affiliations:** 1 Department of Nephrology and Hypertension, Fukushima Medical University, 1, Hikariga-oka, Fukushima, 960-1295 Japan; 2 Department of Pathology, Jusendo General Hospital, Koriyama, Japan

**Keywords:** Anti-glomerular basement membrane nephritis, Dupilumab, Case report, Eosinophilic granulomatosis with polyangiitis, Rapidly progressive glomerulonephritis

## Abstract

**Background:**

Dupilumab, a human monoclonal immunoglobulin G (IgG) antibody that targets the IL-4 and IL-13 receptors, is used to treat various allergic diseases. Several cases of dupilumab-associated autoimmune diseases have been reported in recent years, however, there have been no reports of anti-glomerular basement membrane (GBM) nephritis.

**Case presentation:**

A 54-year-old woman had been receiving treatment for eosinophilic granulomatosis with polyangiitis (EGPA) since the age of 31. At the age of 51, due to worsening asthma symptoms and eosinophilic sinusitis, treatment with dupilumab was initiated. She was later admitted to the hospital with fever and rapidly progressive kidney dysfunction. Glucocorticoid therapy was started because the possibility of recurrence of EGPA was considered. However, her kidney dysfunction progressed without response, and she was transferred to our hospital. Laboratory tests on admission showed positive anti-GBM antibodies, and a kidney biopsy showed crescentic glomerulonephritis with linear IgG deposition along the GBM without findings suggesting the recurrence of EGPA, including eosinophilic infiltration, granuloma formation, or necrotizing vasculitis. Based on her laboratory data and kidney pathological findings, anti-GBM nephritis was diagnosed. Plasma exchange was performed a total of seven times. Although the anti-GBM antibody titer was decreased after the plasma exchanges, kidney function did not improve well. After additional treatment with intravenous cyclophosphamide, her kidney function improved, and she was discharged home.

**Conclusion:**

Anti-GBM nephritis associated with EGPA is extremely rare, and dupilumab may have contributed to its development. In patients receiving dupilumab who develop rapidly progressive kidney dysfunction, the possibility of anti-GBM nephritis should be considered, and prompt diagnosis and therapeutic intervention are essential.

## Background

Anti-glomerular basement membrane (GBM) nephritis is a form of rapidly progressive glomerulonephritis (RPGN) caused by autoantibodies against the GBM. This disease was first reported by Goodpasture in 1919 [1]. The pathogenesis involves autoantibodies (anti-GBM antibodies) directed against the α3 chain of type IV collagen in the GBM. These antibodies are produced due to various causes, and multiple reports suggest that pre-existing nephritis may trigger antibody production [2–5]. Serological tests frequently detect anti-GBM antibodies, and about 30% of patients also test positive for anti-neutrophil cytoplasmic antibodies (ANCAs).

Eosinophilic granulomatosis with polyangiitis (EGPA), formerly known as Churg-Strauss syndrome, is a systemic vasculitis first reported by Jacob Churg and Lotte Strauss in 1951 [6]. EGPA is typically preceded by severe bronchial asthma with eosinophilia and eosinophilic sinusitis. Subsequently, it manifests with two pathological features: eosinophilic inflammation in various organs and vasculitis symptoms, accompanied by a marked increase in the peripheral blood eosinophil count [7, 8]. EGPA has a relatively low myeloperoxidase-ANCA positivity rate of 30–40%, with fewer cases of severe kidney involvement and more frequent pulmonary manifestations, although EGPA is one of the ANCA-associated vasculitis, along with microscopic polyangiitis and granulomatosis with polyangiitis. It is also known that the affected organs differ between ANCA-positive and ANCA-negative cases. ANCA-positive patients are more likely to present with vasculitis-related manifestations such as kidney involvement and peripheral neuropathy. In contrast, ANCA-negative cases more frequently exhibit pulmonary complications and cardiac involvement, with eosinophilic inflammation being the predominant pathological feature [9]. In cases of ANCA-positive RPGN, the presence of anti-GBM antibodies is not uncommon, with Japanese data reporting a prevalence of 5% [10]. However, anti-GBM antibody positivity in ANCA-negative EGPA patients is extremely rare.

Dupilumab, a human monoclonal immunoglobulin G (IgG) antibody that targets the IL-4 and IL-13 receptors, is used to treat various allergic diseases. Although several cases of dupilumab-associated autoimmune diseases have been reported, there have been no reports of anti-GBM nephritis. In this paper, a case of anti-GBM nephritis that developed in a patient with ANCA-negative EGPA during dupilumab therapy is reported along with a literature review.

## Case presentation

A 54-year-old Japanese woman was referred to our hospital for evaluation and treatment of RPGN. She was a non-smoker and had a history of bronchial asthma diagnosed at the age of 28. At 31, she was diagnosed with EGPA based on severe bronchial asthma with eosinophilia, purpura, and gastrointestinal symptoms. Although no skin biopsy was performed, these symptoms improved with glucocorticoid therapy. Eosinophilia persisted after discontinuation of glucocorticoids, but no EGPA relapse was observed. At the age of 51, due to worsening bronchial asthma and eosinophilic sinusitis without other signs of active EGPA despite a prior history, treatment was initiated with dupilumab, an anti–IL-4/IL-13 receptor monoclonal antibody, rather than an IL-5 inhibitor. While on this treatment, she developed fever and RPGN, which led to hospitalization at another facility. Because a relapse of EGPA was suspected, glucocorticoid therapy including methylprednisolone pulse therapy was initiated at the previous hospital. However, her kidney function continued to deteriorate despite treatment, prompting her transfer to our hospital. No treatments other than systemic glucocorticoids had been administered for EGPA.

On admission, the patient’s height was 160.2 cm, and weight was 54.4 kg. Vital signs included blood pressure of 134/84 mmHg, regular heart rate of 77 beats per minute, and temperature of 37.2 °C. On physical examination, there was pitting edema of both lower legs, but no purpura or joint pain. Breath sounds were clear, no heart murmurs were auscultated, and abdominal examination was unremarkable. Laboratory tests showed negative urinary protein but 3 + occult blood with granular casts, suggesting glomerulonephritis. Blood tests showed elevated serum creatinine at 3.56 mg/dL. Serum complement levels were not decreased, and tests for antinuclear antibodies (indirect immunofluorescence titer < 1:160; institutional cutoff ≥ 1:160) and anti-double-stranded DNA antibodies were negative. Though myeloperoxidase-ANCA and proteinase3-ANCA were negative, anti-GBM antibody was markedly elevated at 309 U/mL (Table 1). Chest and abdominal computed tomography revealed no soft tissue density in the paranasal sinuses, but showed diffuse bronchial wall thickening suggestive of chronic airway remodeling from asthma and prior EGPA. Post-inflammatory scarring was present in the lung fields without evidence of active pneumonia. Both kidneys were enlarged.

**Table 1:** Laboratory findings on admission

Urinalysis				Hematology		
pH	5.0			WBC	8,500	/µL
Protein	−			Neu	97	%
Glucose	−			Eos	0	%
Occult blood	3+			Bas	0	%
Urine sediment				Lym	2	%
RBC	30–49	/HPF		Mon	1	%
WBC	1–4	/HPF		RBC	354 × 10^4^	/µL
Granular casts	+			Hb	9.5	g/dL
Oval fat body	−			Ht	29.6	%
Waxy casts	−			Plt	12.1 × 10^4^	/µL
Protein/Cre	0.81	g/gCr				
Biochemistry				Serology		
TP	6.0	g/dL		CRP	0.03	mg/dL
Alb	3.2	g/dL		BNP	44.2	pg/mL
AST	12	IU/L		IgG	1143	mg/dL
ALT	19	IU/L		IgA	101	mg/dL
ALP	92	IU/L		IgM	108	mg/dL
LDH	189	IU/L		IgE	11	mg/dL
CK	19	IU/L		C3	74	mg/dL
UN	61	mg/dL		C4	26	mg/dL
Cre	3.56	mg/dL		CH50	53.1	U/mL
Na	131	mEq/L		ANA	<1:160*	
K	4.8	mEq/L		Anti-dsDNA Ab	0.6	IU/mL
Cl	103	mEq/L		MPO-ANCA	<0.2	U/mL
Ca	8.3	mg/dL		PR3-ANCA	<0.6	U/mL
P	3.3	mg/dL		Anti-GBM Ab	309	U/mL

pH; potential hydrogen, WBC; white blood cell, Neu; neutrophils, Eos; eosinophils, Baso; basophils, Lym; lymphocytes, Mon; monocytes, RBC; red blood cell, Hb; hemoglobin, Hct; hematocrit, Plt; platelet, TP; total protein, Alb; albumin, AST; aspartate aminotransferase, ALT; alanine aminotransferase, ALP; alkaline phosphatase, LDH; lactate dehydrogenase, CK; creatine kinase, UN; urea nitrogen, Cre; creatinine, Na; sodium, K; potassium, Cl; chlorine, Ca; calcium, P; phosphorus, CRP; C-reactive protein, BNP; Brain Natriuretic Peptide, IgG; immunoglobulin G, IgA; immunoglobulin A, IgM; immunoglobulin M, IgE; immunoglobulin E, C3; complement 3, C4; complement 4, CH50; 50% hemolytic complement activity, ANA; antinuclear antibody, myeloperoxidase-anti-neutrophil cytoplasmic antibodies, anti-dsDNA Ab; anti-double stranded DNA antibodies, MPO-ANCA; myeloperoxidase antineutrophil cytoplasmic antibodies, PR3-ANCA; proteinase-3-anti-neutrophil cytoplasmic antibodies, anti-GBM Ab; anti-glomerular basement membrane antibodies. * Institutional cutoff ≥ 1:160

**Fig. 1:**
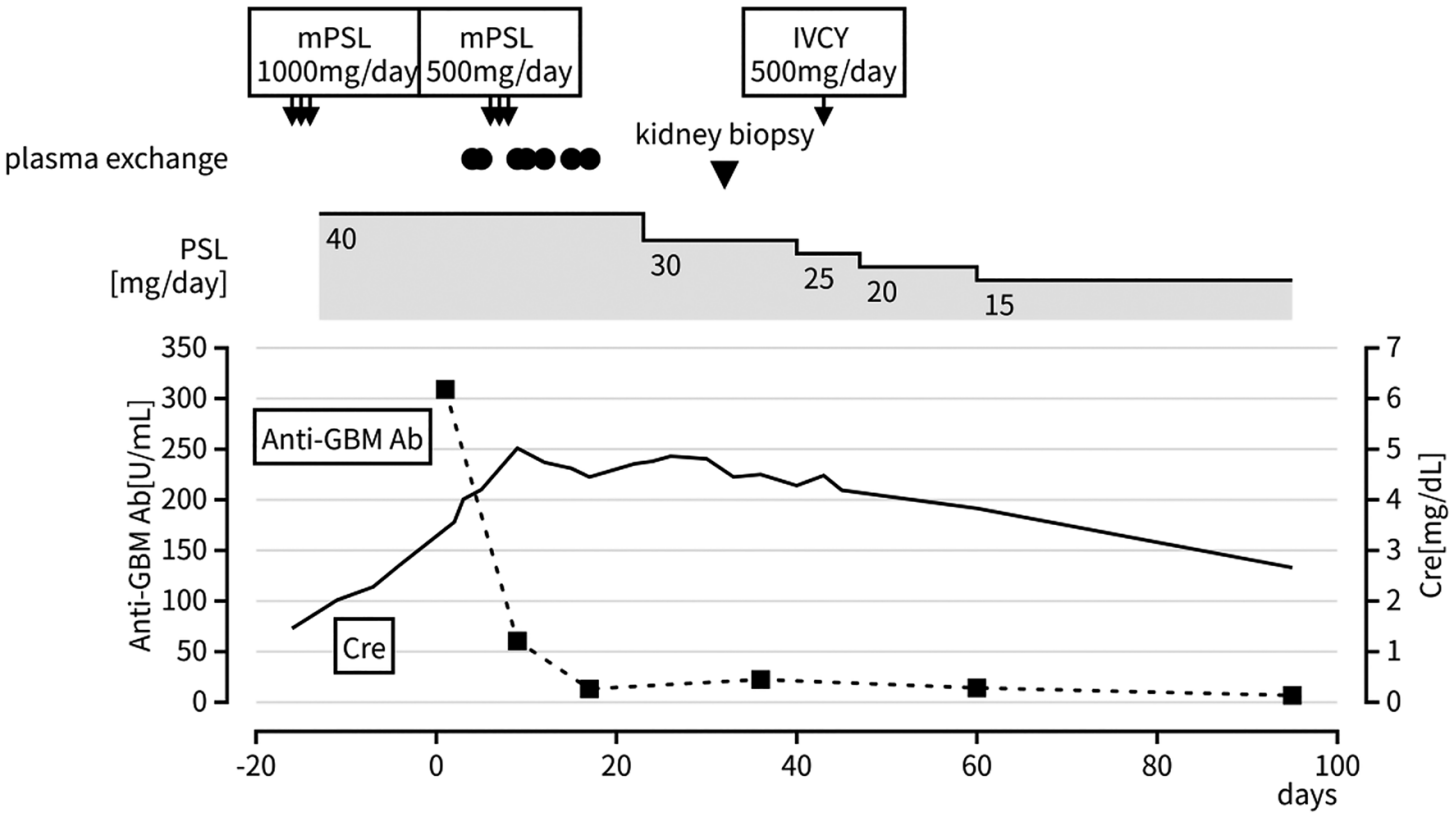
Clinical course of the patient. mPSL (methylprednisolone), PSL (prednisolone), IVCY (intravenous cyclophosphamide therapy), Cre (creatinine), Anti-GBM Ab (anti-glomerular basement membrane antibody)

The clinical course after admission is shown in Fig. 1. The patient continued oral prednisolone (PSL) 40 mg/day but discontinued dupilumab after transfer to our department. Since the anti-GBM antibody test was positive on hospital day 3, suggesting a diagnosis of anti-GBM nephritis, plasma exchange therapy was started on day 4 and performed seven times in total. After the seventh session, the anti-GBM antibody level decreased to 13.2 U/mL, indicating serological improvement. On day 6, methylprednisolone (mPSL) pulse therapy at 500 mg/day was administered for three days concurrently with plasma exchange. Subsequently, oral PSL 40 mg/day was resumed on day 9 and tapered gradually. Despite these initial treatments, kidney dysfunction persisted. A kidney biopsy was performed on day 32.

**Fig. 2:**
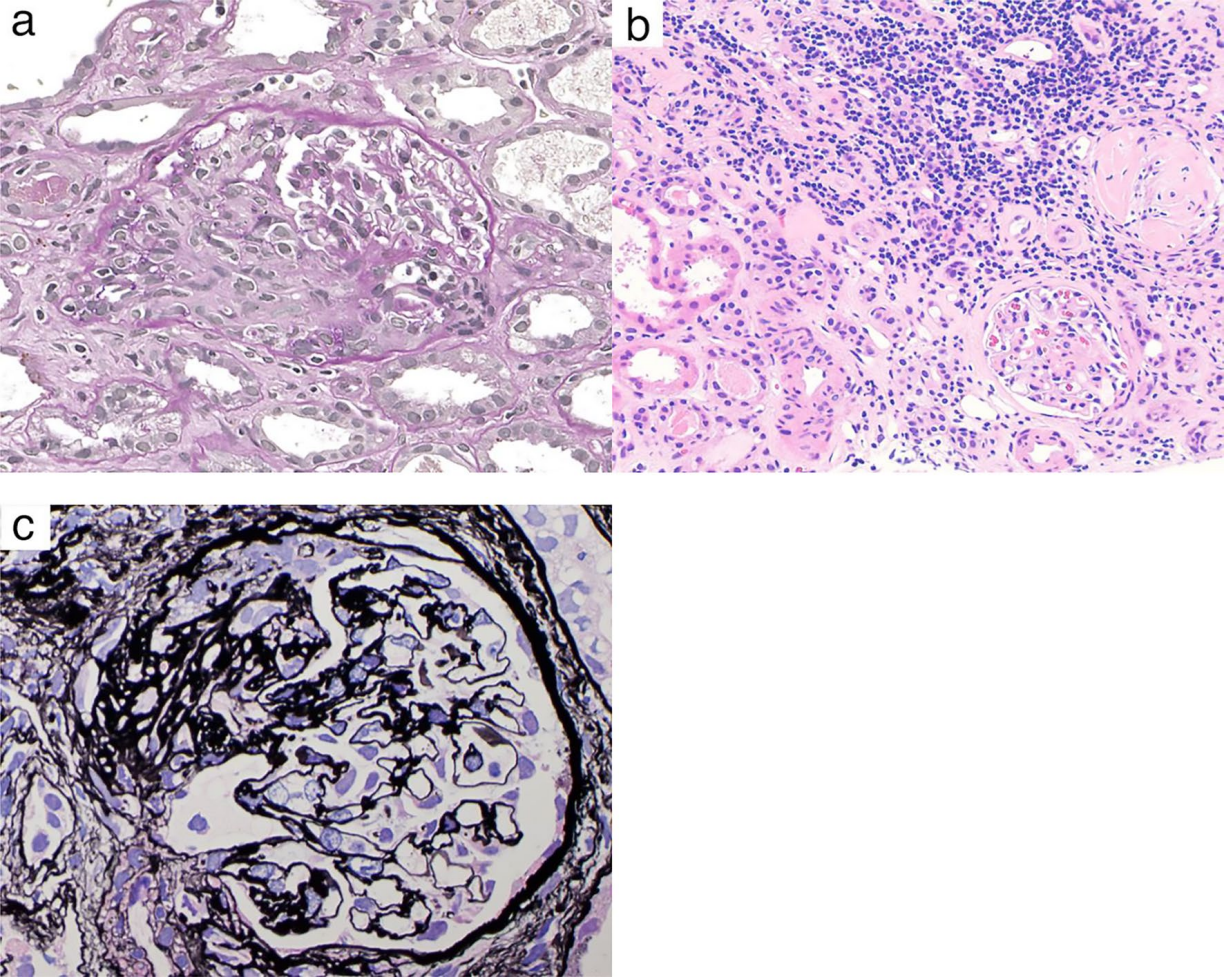
Histological findings. **a** Light microscopy shows fibro-cellular crescents in the glomeruli (PAS stain, original magnification ×400). **b** Lymphocytic infiltration is observed in the tubulointerstitium, but no eosinophilic infiltration is present. No intimal fibrinoid necrosis is seen in the interstitial arteries (HE stain, original magnification ×100). **c** There are no basement membrane lesions, such as spike formation or membrane duplication. (PAM stain, original magnification ×400)

**Fig. 3:**
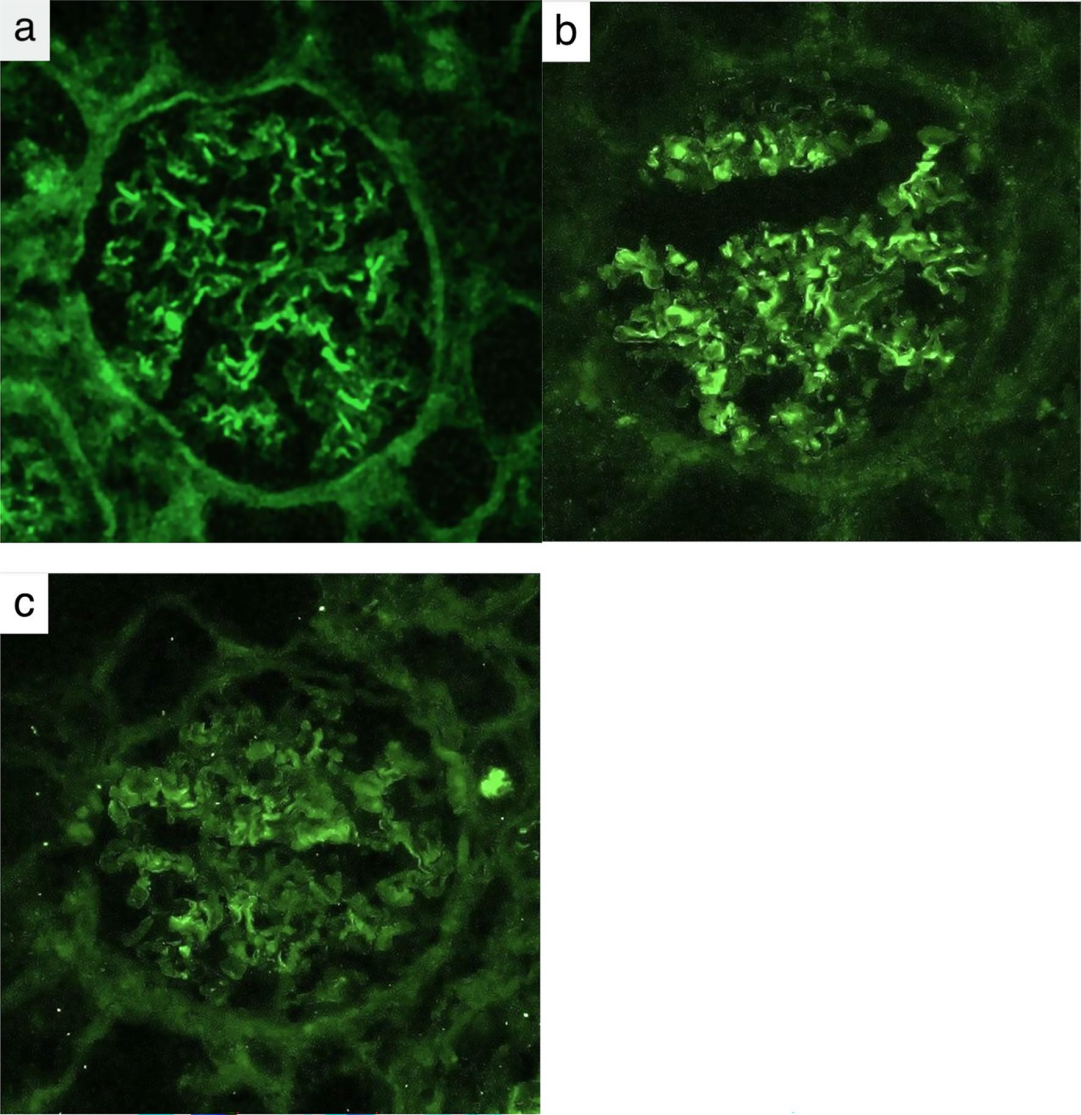
Immunofluorescence study for IgG and IgG subclasses of a kidney biopsy specimen (**a** IgG, **b** IgG1, **c** IgG3). Linear deposits of IgG, IgG1, and IgG3 are observed along the glomerular basement membrane. IgG2 and IgG4 are negative

Pathological findings from the kidney biopsy specimen were as follows. Light microscopy examination of 25 glomeruli showed global sclerosis in 10 glomeruli (40%), fibro-cellular crescent formation in 5 glomeruli (20%), and the remaining 10 glomeruli (40%) were nearly normal. (Fig. 2a). Lymphocytic infiltration was present in the tubulointerstitium, but no eosinophilic infiltration was observed (Fig. 2b). No intimal fibrinoid necrosis was seen in the interstitial arteries. The extraglomerular tissues showed no fibrosis or neovascularization. No necrotic changes with fibrin deposition or endocapillary proliferative changes were observed. Periodic acid-methenamine-silver staining did not show basement membrane abnormalities such as spike formation or doubling of the basement membrane (Fig. 2c). Immunofluorescence studies were negative for complement components (C3, C4, C1q), fibrinogen, and IgA and IgM. IgG was weakly deposited in a linear pattern along the GBM (Fig. 3a). IgG subclass analysis showed predominant deposition of IgG1 over IgG3 (Fig. 3b, c), whereas IgG2 and IgG4 were negative. Electron microscopy demonstrated thickening of the GBM dense layer in some areas, but no definite immune complex deposits were identified.

Based on these histopathological findings, the patient was diagnosed with anti-GBM nephritis. No evidence of eosinophilic infiltration or vasculitis suggestive of EGPA was found. The presence of fibro-cellular crescents in 20% of the sampled glomeruli indicated high disease activity. Consequently, a single intravenous dose of cyclophosphamide 500 mg was added to the treatment regimen on day 43. Following the additional treatment, kidney function showed signs of improvement, and the patient was discharged home on day 48. The post-discharge plan included gradual tapering of glucocorticoid in the outpatient setting. On day 95, laboratory tests showed that the serum creatinine had improved to 2.6 mg/dL with no re-elevation of anti-GBM antibodies. She was subsequently transferred back to the referring hospital. Notably, hemodialysis was not required throughout the clinical course.

## Discussion and conclusions

This case report describes anti-GBM nephritis that developed in a patient with EGPA during dupilumab therapy. The patient had been diagnosed with EGPA 23 years earlier, following the presentation of severe bronchial asthma with eosinophilia, purpura, and gastrointestinal symptoms. Although peripheral eosinophilia persisted after the discontinuation of steroid therapy, there was no evidence of EGPA exacerbation. During the clinical course, dupilumab was administered for the treatment of eosinophilic asthma and sinusitis. At the current presentation, the patient developed acute kidney injury, and laboratory testing revealed elevated levels of anti-GBM antibodies. Plasma exchange and glucocorticoid pulse therapy were initiated; however, improvement in kidney function was limited. Kidney biopsy demonstrated linear IgG deposition along the GBM without eosinophilic infiltration or granuloma formation. Based on these findings, a diagnosis of anti-GBM nephritis was confirmed, and intravenous cyclophosphamide was added to the treatment regimen, leading to improvement in kidney function.

In general, in ANCA-associated vasculitis and anti-GBM nephritis, kidney prognosis is known to correlate with the proportion of normal glomeruli observed on biopsy and the serum creatinine level at the initiation of treatment. In anti-GBM nephritis, a proportion of normal glomeruli below 10% has been reported to be associated with poor kidney outcomes [11, 12]. In the present case, 10 out of 25 glomeruli (40%) were histologically normal, which may have contributed to the avoidance of end-stage kidney disease. Additionally, the patient’s serum creatinine level was 3.56 mg/dL at the time of treatment initiation, suggesting that early therapeutic intervention also played a role in the favorable kidney outcome. Furthermore, the observation that kidney involvement appears milder than in typical anti–GBM nephritis may represent a characteristic feature of drug-induced anti–GBM disease; as additional cases are accumulated, its clinicopathologic features should be more clearly defined.

Anti-GBM nephritis is a disease characterized serologically by positive anti-GBM antibodies. Histologically, it typically presents as necrotizing crescentic glomerulonephritis [13]. This disease is extremely rare, with an estimated 100 new cases occurring annually in Japan [14]. Reported triggers include infections, inhalation of toxic substances, smoking, and ANCA-associated vasculitis. Pathophysiologically, continuous damage to the pulmonary and kidney basement membranes leads to exposure of antigenic epitopes embedded within the basement membrane. Anti-GBM antibodies are produced against these exposed antigenic epitopes [15, 16]. Glomerulonephritis develops when these antibodies deposit on the GBM of the kidney.

EGPA is a vasculitis characterized by peripheral blood eosinophilia, with preceding symptoms of bronchial asthma and rhinosinusitis. It is also extremely rare, with an incidence of 0.5–4.2 per million persons annually [17]. The clinical manifestations are diverse, including peripheral neuritis; purpura; gastrointestinal involvement—such as eosinophilic gastroenteritis, abdominal pain, and diarrhea; eosinophilic myocarditis; pericarditis; and kidney dysfunction. The pathophysiology of EGPA has a dual nature of eosinophilic inflammation and necrotizing vasculitis. IL-5 and ANCA are involved in its pathogenesis. IL-5 promotes eosinophil differentiation and maturation, inducing tissue damage through cytotoxic granular proteins and lipid mediators. Meanwhile, ANCA activates neutrophils, causing vasculitis by promoting degranulation of cytotoxic factors and formation of neutrophil extracellular traps. There are differences in the distribution of organ damage between ANCA-negative and ANCA-positive cases. In general, kidney involvement is less frequently observed in ANCA-negative cases, as in the present case [9, 18]. In fact, no histological findings suggestive of EGPA-related kidney damage were observed in the present case, although immunosuppressive therapy including glucocorticoid pulse therapy had been administered prior to the kidney biopsy, and histological findings derived from EGPA, such as eosinophilic infiltration or necrotizing vasculitis, might have weakened and disappeared due to treatment.

Reports of anti-GBM nephritis associated with ANCA-associated vasculitis due to microscopic polyangiitis or granulomatosis with polyangiitis are occasionally found, however, the present literature search identified only two reported cases of anti-GBM nephritis associated with EGPA [19, 20]. Among these cases, only one case was ANCA-negative. Matsuzaki et al. [19] reported a case of ANCA-negative EGPA with positive anti-GBM antibodies. This case presented with multiple joint pain, anemia, hypoxemia, mononeuritis multiplex, and kidney dysfunction. During the course of the disease, anti-GBM antibodies became positive, and open lung biopsy showed granuloma formation and vasculitis with severe eosinophilic infiltration, leading to a diagnosis of EGPA. In this case report, it is speculated that strong eosinophilic infiltration caused extensive pulmonary damage, leading to exposure and injury of the airway mucosa and alveolar basement membrane. The resulting exposure of the alveolar basement membrane may have triggered the production of anti-GBM antibodies through shared antigenicity. Recently, an autoantibody against laminin-521, which has been linked to pulmonary damage and smoking, was reported to be associated with the development of anti-GBM nephritis [21]. This autoantibody is predominantly of the IgG1 and IgG4 subclasses. In our case, although information regarding passive smoke exposure was uncertain, the patient was a never-smoker, and kidney histology showed predominant deposition of IgG1 and IgG3 in the IgG subclass analysis. Therefore, the likelihood that anti-GBM antibodies were produced as a result of airway mucosal injury due to pulmonary damage appears to be low in this case, although marked eosinophilia was observed.

Dupilumab is a recombinant human IgG4 monoclonal antibody that specifically binds to the IL-4 receptor alpha subunit, which is shared by the receptor complexes for human IL-4 and IL-13, thereby inhibiting the signaling of both IL-4 and IL-13 [22, 23]. It is used in the clinical treatment for atopic dermatitis, uncontrolled asthma, and eosinophilic sinusitis. There have been several reports of the development of autoimmune diseases characterized by Th1-dominant or mixed Th1/Th2 immune responses—such as type 1 diabetes, rheumatoid arthritis, painless thyroiditis, and systemic lupus erythematosus (SLE)—following dupilumab administration [24–27]. In the field of nephrology, the onset or exacerbation of IgA nephropathy has also been reported [28]. Several cases of EGPA occurring after dupilumab have been reported recently as well [29–31]. These cases suggest that suppression of Th2-related cytokines by dupilumab may lead to a relative dominance of Th1 responses, potentially disrupting immune tolerance. Anti-GBM nephritis is known to be mediated primarily by Th1-type immune responses, and the pathogenic antibodies are predominantly of the IgG1 and IgG3 subclasses [32]. In the present case as well, IgG1 and IgG3 were deposited along the glomerular basement membrane, while IgG4, which is typically associated with Th2 responses, was not detected. Similar to previous reports, it is possible that dupilumab suppressed Th2 responses and induced a Th1-skewed immune reaction, thereby contributing to the development of anti-GBM nephritis in our patient, although it took 3 years after the initiation of dupilumab. Based on our literature review, there have been no reports of dupilumab-associated anti-GBM nephritis. The accumulation of similar cases in the future may help elucidate the pathogenic mechanism of anti-GBM nephritis induced by dupilumab.

We report a case of anti-GBM nephritis that developed in a patient with EGPA who was receiving dupilumab. Dupilumab is widely used in clinical practice for the treatment of various allergic diseases. Clinicians should be aware of the potential development of dupilumab-associated autoimmune diseases. In particular, when RPGN occurs in patients receiving dupilumab, the possibility of anti-GBM nephritis should be considered. Therefore, histological evaluation by kidney biopsy is essential in such cases to identify the underlying cause of kidney dysfunction, and appropriate therapeutic interventions should be implemented based on the pathological findings.

## Data Availability

All data generated or analyzed during this study are included in this published article. No additional datasets were generated.

## Abbreviations

GBM: Glomerular basement membrane
RPGN: Rapidly progressive glomerulonephritis
ANCA: Anti-neutrophil cytoplasmic antibody
EGPA: Eosinophilic granulomatosis with polyangiitis
